# Hemophagocytic Lymphohistiocytosis Presenting With ARDS in a Young Adult: A Case Report

**DOI:** 10.1155/2024/1266606

**Published:** 2024-11-07

**Authors:** Zehra Dhanani, Stephen Dachert

**Affiliations:** ^1^Thoracic Medicine and Surgery, Temple University Hospital, Philadelphia, Pennsylvania, USA; ^2^Thoracic Medicine and Surgery, Lewis Katz School of Medicine at Temple University, Philadelphia, Pennsylvania, USA

**Keywords:** ARDS, case report, febrile illness, intensive care unit

## Abstract

Fever is common in the ICU, with infectious causes accounting for only half of febrile episodes. This case examines a young male who developed high-grade fevers and pulmonary infiltrates unresponsive to broad-spectrum antibiotics. Examination revealed hepatosplenomegaly, hypertriglyceridemia, anemia, and thrombocytopenia, suggestive of hemophagocytic lymphohistiocytosis (HLH). Meeting 5 of 8 HLH criteria, high-dose steroids were administered, resulting in clinical improvement. HLH, with a high mortality risk, demands early recognition, complicated by nonspecific symptoms. This case highlights the rare manifestation of ARDS in HLH, adding diagnostic challenges in critical care settings.

## 1. Introduction

Fever is one of the most common clinical signs in the intensive care unit (ICU) which can signify numerous different pathologies. The prevalence of fever in the ICU patients ranges from 26% to 70%, with nearly equal distribution between infectious and noninfectious etiologies [1, 2]. However, physician bias toward infectious etiologies often leads to unnecessary antibiotic use and potentially overlooking critical noninfectious diagnoses [2]. Here, we present a case of a young male with persistent fever, despite antibiotic administration, serving as a compelling diagnostic challenge.

## 2. Case Report

A 20-year-old Caucasian male with no prior medical or surgical history presented with 5 days of intractable vomiting. In the emergency department, he experienced multiple seizures, necessitating intubation. Examination revealed tachycardia and dry mucus membranes. Laboratory data demonstrated metabolic alkalosis, acute kidney injury, and a positive urine drug screen for cannabis. Management was initiated for presumed cannabis-induced cyclic emesis.

On the fifth day of admission, the patient developed high-grade fevers and evolving bilateral pulmonary infiltrates. Broad-spectrum antibiotics were started with vancomycin and piperacillin-tazobactam. Bronchial aspirates grew methicillin-sensitive *Staphylococcus aureus* and *Escherichia coli,* both sensitive to the initial antibiotics. Despite appropriate antibiotic administration, the patient continued to have fevers that persisted for 10 days, 5 days of which occurred while on broad-spectrum antibiotics, with a maximum temperature of 39.5°C. Thorough examination and imaging did not identify other infectious or noninfectious sources.

**Figure 1:**
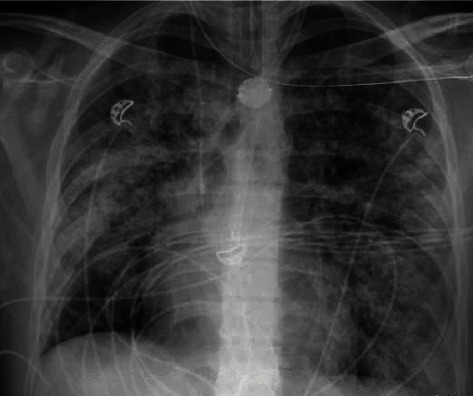
Chest x-ray.

Due to persistent fevers, antibiotics were escalated to meropenem, and the antifungal agent micafungin was added. However, these adjustments did not improve the fever curve. The patient's hypoxemia worsened, leading to acute respiratory distress syndrome (ARDS) requiring venovenous extracorporeal membrane oxygenation (VV-ECMO) support (Figure 1).

**Figure 2:**
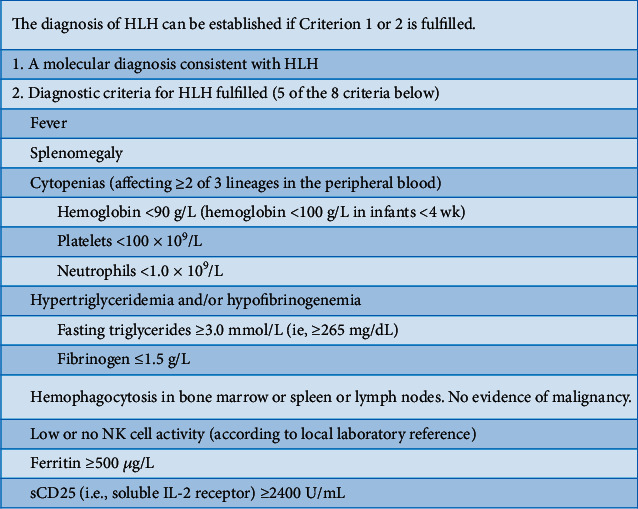
The HLH 2004 Diagnostic Criteria [3].

A review of the patient's chart revealed evidence of hepatosplenomegaly on imaging (though not detected on physical examination), hypertriglyceridemia (379 mg/dL), anemia (6.4 g/dL), and thrombocytopenia (88 K/mm^3^) with a normal white blood cell and neutrophil count. Ferritin levels were elevated (1188 ng/mL), raising suspicion for hemophagocytic lymphohistiocytosis (HLH). With 5 out of 8 HLH criteria met and an “H score” of 170, high-dose steroids were initiated (Figure 2). The patient was started on dexamethasone at 10 mg/m^2^, tapered over 8 weeks. Soluble CD25 level was elevated to 2204, although below the cut-off of 2400 for HLH, which may have been affected by prior steroid administration. A bone marrow biopsy was considered but not performed due to patient's critical condition and extracorporeal membrane oxygenation (ECMO) cannulation limiting positioning.

**Figure 3:**
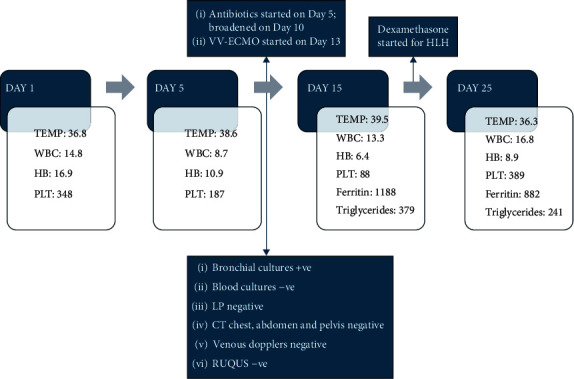
Sequence of events. WBC, white blood cell count; HB, hemoglobin; PLT, platelet count; VV-ECMO, venovenous extracorporeal membrane oxygenation; LP, lumbar puncture; RUQUS, right upper quadrant ultrasound.

The patient showed clinical improvement with steroids and was decannulated from ECMO, extubated, and discharged on a steroid taper to acute rehab.  Figure 3 summarizes the sequence of events.

## 3. Discussion

HLH, with reported mortality of 20% to 88%, was traditionally considered a disease of childhood, but is now increasingly recognized in adults [4]. Abdelhay et al.'s review of over 16,000 patients revealed that HLH in adults tends to occur most commonly in Caucasian males between the ages of 16–30 and 56–70 [5]. In adults, HLH is frequently linked to infections, primarily viral and less commonly bacterial, as well as hematological malignancies, notably lymphoma, and autoimmune diseases [6]. Our case is most consistent with secondary HLH in the context of a pulmonary infection, although rare cases of primary HLH can present in adulthood [7]. The uniqueness of our patient's presentation lies in the development of ARDS, an uncommon manifestation of HLH. ARDS in the context of HLH is exceptionally rare, with only a limited number of documented case reports in the literature [8, 9].

The HLH-2004 diagnostic criteria, derived from the HLH trial, remains the cornerstone of diagnosis [10]. However, it is important to note that many components of the criteria include nonspecific signs and symptoms that can be easily overlooked or misattributed to other etiologies as seen in our case, where high-grade fever and cytopenias were initially attributed to sepsis and hypertriglyceridemia to propofol use [4]. Notably, the evidence of hepatosplenomegaly was identified through imaging but not during the physical examination, illustrating an example of anchoring bias, where clinicians may fixate on a primary diagnosis—in this case, a pulmonary infection—and overlook other significant findings that could indicate a different underlying condition. This emphasizes the necessity for a heightened index of suspicion, especially for a rare disorder like HLH that may not commonly present in clinical practice.

Importantly, the HLH-2004 diagnostic criteria was originally formulated for research purposes. While external validity studies indicate test sensitivity and specificity in the range of 90%, it is not without its limitations [11]. One such limitation is the incapacity of many centers to perform advanced tests, such as soluble CD25 level, which frequently require outsourcing to other laboratories resulting in a turnaround time of at least 3–4 days. Due to the high mortality associated with HLH, treatment is often initiated before obtaining results of all the necessary laboratory data. The situation is further complicated by the unclear impact of steroids on CD25 levels, as observed in our patient who received dexamethasone before CD25 level measurement. Furthermore, the requirement for a bone marrow biopsy in the diagnostic criteria poses a challenge, as this procedure is not routinely performed by critical care physicians and can be difficult to obtain in critically ill patients like ours. Emerging data recommends adoption of modified criteria for HLH diagnosis in adults, allowing for a more inclusive and lenient approach [12].

In summary, our case delineates a unique presentation of a rare disease, serving as an excellent illustration of anchoring bias and the need for an expansive differential diagnosis. Additionally, this case sheds light on the need and validation of more clinically oriented scoring systems for diagnosis of HLH.

## Data Availability

Data sharing is not applicable to this article as no new data were created or analyzed in this study.
